# Biostasis: A Roadmap for Research in Preservation and Potential Revival of Humans

**DOI:** 10.3390/brainsci14090942

**Published:** 2024-09-21

**Authors:** Andrew T. McKenzie, Brian Wowk, Anton Arkhipov, Borys Wróbel, Nathan Cheng, Emil F. Kendziorra

**Affiliations:** 1 Apex Neuroscience, Salem, OR 97317, USA; 2 21st Century Medicine, Inc., Fontana, CA 92336, USA; 3 Allen Institute, Seattle, WA 98109, USA; 4 European Institute for Brain Research, 1181LE Amstelveen, The Netherlands; 5 BioPreservation Institute, Vancouver, WA 98661, USA; 6 Longevity Biotech Fellowship, San Francisco, CA 95050, USA; 7 European Biostasis Foundation, 8197 Rafz, Switzerland

**Keywords:** aldehyde-stabilized cryopreservation, biostasis, brain preservation, cryonics, cryopreservation, vitrification, chemical fixation, connectomics, molecular nanotechnology, whole-brain emulation

## Abstract

Human biostasis, the preservation of a human when all other contemporary options for extension of quality life are exhausted, offers the speculative potential for survival via continuation of life in the future. While provably reversible preservation, also known as suspended animation, is not yet possible for humans, the primary justification for contemporary biostasis is the preservation of the brain, which is broadly considered the seat of memories, personality, and identity. By preserving the information contained within the brain’s structures, it may be possible to resuscitate a healthy whole individual using advanced future technologies. There are numerous challenges in biostasis, including inadequacies in current preservation techniques, methods to evaluate the quality of preservation, and potential future revival technologies. In this report, we describe a roadmap that attempts to delineate research directions that could improve the field of biostasis, focusing on optimizing preservation protocols and establishing metrics for querying preservation quality, as well as pre- and post-cardiac arrest factors, stabilization strategies, and methods for long-term preservation. We acknowledge the highly theoretical nature of future revival technologies and the importance of achieving high-fidelity brain preservation to maximize the potential of future repair technologies. We plan to update the research roadmap biennially. Our goal is to encourage multidisciplinary communication and collaboration in this field.

## 1. Introduction

Biostasis is the practice of preservation of humans for the long-term with the intent of future recovery, if this ever becomes feasible. Biostasis can be distinguished into two hypothetical modalities: (a) provably reversible preservation and (b) preservation of informational features in the body in a way that is not reversible with currently known technologies, with the hope that such technologies can be developed and implemented in the future [1,2,3,4]. Provably reversible preservation, also known as suspended animation, is not yet possible for humans, and probably will not be possible anytime soon, absent incredibly rapid advances in preservation technology. Yet, contemporary biostasis methods do not need to be proved to be reversible now in order to allow for a potential chance at revival in the future. The primary justification of contemporary biostasis, which we adopt here, is the preservation of the brain, which most consider to be the seat of our memories, personalities, and identities [2,5]. By preserving the information contained within the structures of the brain, we may one day be able to revive the individual using advanced future technologies, even though this would require society to bootstrap the development of those technologies while individuals remain under preservation. This practice is also called neural biostasis, brain preservation, or brain archiving. We use the more general term biostasis in this roadmap for three reasons. First, we also discuss technologies that may enable the preservation of more than just the brain. Second, we intend for this roadmap to be updated into the medium-term future, when provably reversible methods may have gained significantly more traction. Finally, many people in the field feel strongly that whole-body preservation is essential for maintenance of personal identity, and we want the research roadmap to be compatible with their perspectives as well [6]. In future versions of the roadmap, we also plan to explore the potential benefits and challenges of whole-body preservation compared to primarily focusing on brain preservation, as we recognize the importance of this topic for many in the biostasis community. We provide a set of key terms used in this manuscript to assist with reader comprehension (Table 1). 

In this publication, we focus on the scientific and technical aspects of biostasis, rather than discussing its historical context or the philosophical debates surrounding its feasibility and desirability. These topics are undoubtedly important and have been extensively discussed in other works [3,7,8,9,10,11,12]. However, a discussion of these topics would be beyond the scope of this research roadmap. Furthermore, we acknowledge that the pursuit of biostasis raises a multitude of ethical and societal questions that must be carefully considered. These issues, ranging from the allocation of resources to the potential impact on social structures and individual autonomy, are complex and multifaceted. Although these ethical and societal implications are of paramount importance, they have been thoroughly explored in other publications dedicated to these specific aspects of biostasis, a few examples of which we refer the interested reader to [2,13,14,15,16]. We brought together multidisciplinary perspectives to frame the major open problems in biostasis research and how they could possibly be addressed. The literature selection for this roadmap was based on an ad hoc approach, leveraging the collective expert knowledge of the authors and other colleagues who we consulted with. While we believe biostasis research holds significant potential, this roadmap aims to impartially assess the current state of the field and identify key areas for future investigation. Our goal is certainly not to suggest that biostasis is a guaranteed solution, but rather to encourage rigorous scientific inquiry that could advance our understanding of long-term biological preservation and potentially lead to breakthroughs in medical science in the future. 

A key distinction in the field of biostasis is between approaches that (a) use exclusively cryopreservation-based approaches and (b) approaches that use aldehyde fixation, with or without cryopreservation as well. We briefly describe these approaches here for context. Pure cryopreservation aims to preserve tissue in a vitrified state at very low temperatures, relying on high concentrations of cryoprotectants to prevent ice crystal formation and maintain cellular structure. The primary challenge with this approach is adequately distributing the cryoprotectants while achieving sufficiently rapid cooling and warming rates to avoid ice formation and also minimizing toxicity from the cryoprotectants themselves. Reversing the damage caused by contemporary cryopreservation approaches will be a major challenge for future revival efforts. In contrast, aldehyde-based approaches use chemical fixatives, such as formaldehyde and glutaraldehyde, to crosslink proteins and stabilize tissue structure. This allows for excellent morphological preservation at the cost of altering the tissue’s biomolecular state. Aldehyde fixation can be done as a standalone procedure, with the fixed tissue stored in a preservative solution at above-freezing temperatures, or it can be combined with cryopreservation for enhanced long-term stability [17]. In the latter case, cryoprotectants are distributed into the fixed brain tissue, which is then cooled to very low temperatures. The crosslinking from the fixatives helps to maintain structural integrity during the cryopreservation process. However, reversing the chemical alterations caused by fixation is a major challenge for future revival efforts. The choice between these broad categories of approaches involves weighing factors such as the desired level of structural preservation, the extent to which cryopreservation and aldehyde fixation each damage tissue, cost, complexity, and assumptions about the capabilities of future revival technologies (Table 2).

We note that while determining what specific structures in the brain need to be preserved for successful future revival is critically important, it is generally outside the scope of this roadmap. Instead, the focus here is on developing and optimizing the preservation methods that can maintain the highest possible fidelity to the original state of the brain, thus maximizing the potential for future restoration under a wide range of possible encoding methods for personal identity that the neuroscience community has discovered so far and may elaborate upon in the future. 

**Figure 1:**
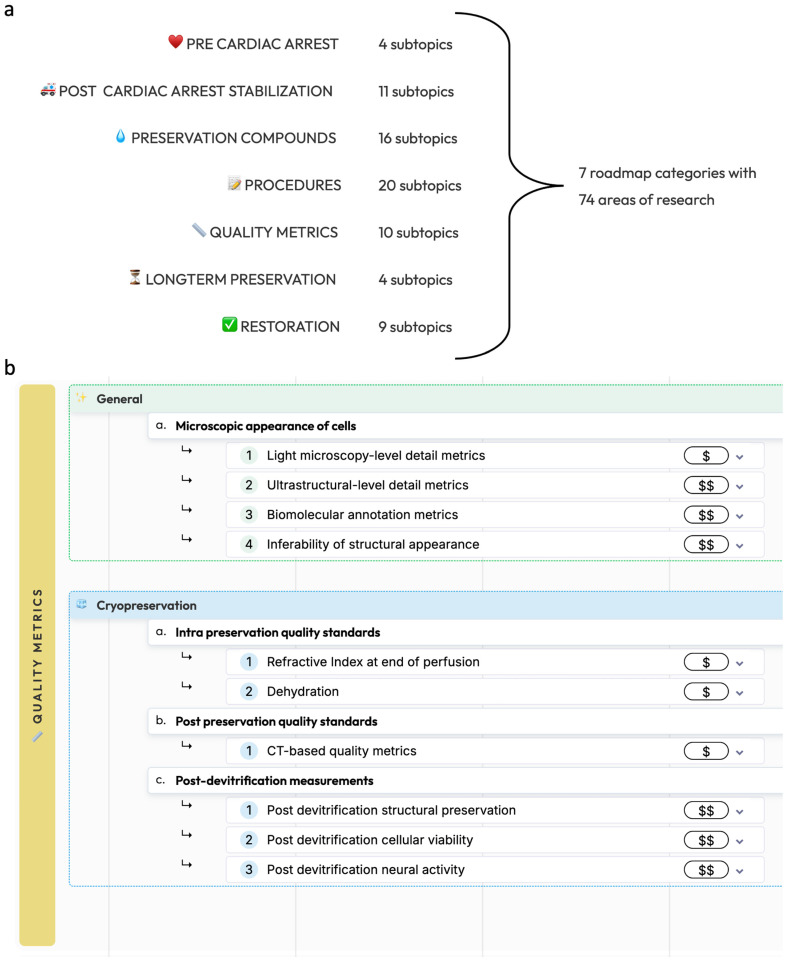
Overview of the biostasis research roadmap. (**a**): The seven main categories of the biostasis research roadmap with the number of subtopics in each. (**b**): As an example, a detailed view of the quality metrics category is shown, showing general and cryopreservation-specific research areas. The full roadmap is available at https://www.biostasis.xyz/ (accessed on 18 July 2024).

This roadmap is divided into seven main categories: pre-cardiac arrest factors, post-cardiac arrest stabilization, preservation compounds, preservation procedures, methods for measuring preservation quality, long-term preservation, and restoration and recovery (Figure 1). Procedures without prior circulatory arrest are beyond the scope of the present roadmap, as it is important to note that legal death accompanied by cardiac arrest is currently a legal requirement for any biostasis procedure. 

## 2. Pre-Cardiac Arrest Factors

This category refers to the various health conditions, medications, and medical interventions that an individual undergoing biostasis may experience prior to cardiac arrest. Studying these factors is crucial for understanding how they may impact the quality of brain preservation. This includes investigating the role of maintaining circulatory system health, with a focus on the most important factors such as hypertension, diabetes, stroke, and general factors of aging. Additionally, researchers can aim to determine how different antithrombotic regimens that individuals are prescribed by their healthcare professionals, including medications like aspirin, heparin, and direct-acting oral anticoagulants, might affect the quality of perfusion and brain preservation. Another area of interest is exploring whether procedural changes based on pre-mortem medical data, such as CT scans, MRIs, and pre-existing conditions, can be beneficial in optimizing brain preservation outcomes. By considering these variables, researchers may be able to develop more tailored or “personalized” preservation protocols that account for them. It is important to note that if pre-cardiac arrest factors are found to significantly impact the quality of brain preservation, this information could also theoretically help people to make informed decisions about their end-of-life care, in consultation with their healthcare providers. However, any interventions or protocol changes based on these findings would need to be carefully implemented by the individual’s healthcare team to ensure they are legal and ethically appropriate within the relevant jurisdictions. Ultimately, the goal of studying pre-cardiac arrest factors is to provide individuals with the knowledge they need to make informed choices about their biostasis arrangements consistent with their values, while ensuring that any actions taken are legally and ethically sound and do not cause undue harm. 

Another important consideration is the possibility of pre-preservation brain damage affecting the structures encoding an individual’s identity, such as their memories and personality, to such an extent that they are no longer available to be preserved. This could occur from conditions such as brain trauma, severe malnutrition, focal cerebral ischemia due to stroke, global cerebral ischemia during cardiac arrest followed by resuscitation and reperfusion injury while on life support, prolonged agonal state, or various types of neurodegenerative disease. Of course, there is enormous variability in these conditions. There is also evidence that some clinically apparent damage can be due to processes that impair functional readout rather than the loss of all underlying crystallized memories, as can be seen in some conditions where there are moments of lucidity [18]. As a result, determining the extent of brain damage that would render biostasis futile is a complex challenge, requiring future research. The limiting case of so much loss of brain structure that a repaired brain and resuscitated person would not be the original person has been called information-theoretic death [1]. 

It is important to distinguish between the effects of neurodegenerative disorders and acute conditions on the brain. Neurodegenerative disorders like Alzheimer’s disease or Parkinson’s disease can cause gradual, progressive loss of neural structures over time, potentially compromising the preservation of aspects of personal identity. In contrast, acute conditions such as stroke or traumatic brain injury can cause rapid, localized damage that may have different implications for preservation quality. A third category is acute changes to brain function due to potentially reversible causes, such as delirium due to hepatic, uremic, or sepsis-associated encephalopathy. These conditions can have substantially different effects on brain structure and may have distinct implications in the context of biostasis. 

This question becomes particularly relevant for individuals and their families who must decide when to withdraw supportive care, including ventilator support during brain injury or other critical illness with poor prognosis, or nutrition and hydration during terminal illness, providing comfort care only. It is also relevant to the various forms of medical aid in dying or euthanasia. The qualifying diseases, remaining life expectancies, and options for physician-initiated or physician-assisted legal death that are available are complex, jurisdiction-dependent, and frequently changing [19]. It is not possible to discuss them all here, except to note that any decision to affect the timing of cardiac arrest and legal death for purposes of biostasis must be made by the individual themselves, based on their own beliefs, values, and unique situation [20]. These decisions are not unique to biostasis. They are faced by individuals and their families in all situations of serious illness and end-of-life care.

From a scientific perspective, it would be useful to establish evidence-based guidelines—to the extent possible—for determining the degree of neurodegeneration that would make a biostasis procedure unlikely to preserve the essential features of the individual’s brain. This could help people to make challenging but informed decisions about when they want to time entry into biostasis. However, it is crucial to emphasize that the topic of timing of legal death for purposes of biostasis requires careful consideration of the ethical implications and respect for individual autonomy in end-of-life decisions. The purpose of discussing this question in the context of the research roadmap is certainly not to encourage particular medical care decisions, but rather to acknowledge the potential for future scientific research that may be relevant to end-of-life care in the context of biostasis.

Finally, another consideration in the pre-cardiac arrest phase is the timely deployment of standby teams that can monitor the condition of someone who is gravely ill and be available to stabilize the individual immediately after the declaration of legal death, for organizations employing this model of care. Determining the optimal timing for deploying these teams is critical, as early intervention can significantly improve the quality of brain preservation, but premature deployment can be costly and potentially disruptive to the individual and their loved ones. Research should focus on developing better methods for assessing when an individual’s health has severely decompensated, indicating an increased likelihood of legal death in the near future. This may involve studying various biomarkers, vital signs, and other clinical indicators that can reliably predict imminent legal death, as well as exploring the potential benefits of integrating advanced monitoring technologies to measure an individual’s health status and alert standby teams when necessary. Of course, any such monitoring must be conducted with the full consent and cooperation of the individual and their healthcare providers and must not interfere at all with the provision of appropriate medical care. 

## 3. Post-Cardiac Arrest Stabilization

Sometimes it may not be possible to begin biostasis procedures until after long periods of clinical death, which results in global cerebral ischemia. This is common in the practice of cryonics, which traditionally follows a “no patient left behind” ethos in which any human remains that can be recovered are cryopreserved because of a reluctance to make a contemporary judgement call about how much brain structure loss erases individual identity [21], especially because the question may be non-binary [22]. A particular problem caused by long ischemia after cardiac arrest is the difficulty or impossibility of perfusing preservation solutions through blood vessels. This requires further research, including the possible introduction of fixatives and cryoprotectants by external diffusion, and accurate determination of the circumstances of when perfusion should be attempted or not. 

In all jurisdictions we are aware of, biostasis teams must not begin any preservation procedures until the patient has been evaluated by a healthcare professional and legal death has been pronounced following cardiac arrest. After this legal pronouncement has been made, stabilization can begin, which focuses on minimizing the deterioration of the brain as soon as possible and preparing the patient for the subsequent definitive preservation procedures. For example, stabilization might be done by a local team prior to and during transport of the body to a facility where a definitive preservation procedure can be performed [23]. The most critical aspect of this, when cardiac arrest is expected, is the positioning of an appropriately trained and equipped team to standby at the patient’s location before cardiac arrest occurs. Only with such a team present can warm ischemia before preservation be limited to a small number of minutes, ischemic injury to the brain be minimized, and the ability to later perfuse the brain with a preservative solution be optimal.

An especially challenging problem—especially for individuals who live alone—is the rapid and reliable determination of unexpected cardiac arrest, in order to notify medical professionals and prevent long periods of unattended global cerebral ischemia. To address this, researchers should focus on developing more accurate and reliable methods for monitoring an individual’s health status. This may involve exploring new technologies and algorithms that can decrease false negatives and false positives. For example, wearable devices coupled with remote monitoring systems could be designed to continuously track vital signs and alert local contacts immediately upon detecting a cessation of cardiac activity. Through these efforts, biostasis organizations can help ensure that emergency medical care is initiated as quickly as possible. If all appropriate medical interventions have failed, biostasis stabilization procedures can be initiated as soon as possible, minimizing the detrimental effects of warm ischemia on the brain. This will often require engagement with local coroners or medical examiners with mandates to investigate unexpected cardiac arrest so that biostasis can be authorized as soon as possible, and so that the brain is not invasively autopsied. This can be facilitated by the patient’s physician and medical history being rapidly accessible to satisfy investigators about the cause and mode of legal death. A critical aspect of legal advocacy in biostasis, beyond the scope of this research roadmap, is to establish the legal right for individuals to have their desire for biostasis respected and implemented in a timely manner, as an extension of their bodily autonomy. Biostasis organizations would ideally be allowed to work with legislators and medical professionals to create policies and procedures that streamline the process of initiating biostasis after legal death, while still ensuring proper investigation and documentation of the cause of legal death when indicated. This is one way in which current medical practice should adapt to the evolving research landscape in this field, to respect the body autonomy of individuals who desire biostasis. We note that death investigation practices vary widely across jurisdictions internationally, necessitating more research on this complex topic. 

A key aspect of post-cardiac arrest stabilization is the administration of a general medication protocol [23], which can include anticoagulants, neuroprotectants, and agents that help minimize cerebral edema. Anticoagulants, such as heparin or sodium citrate, can be used to prevent or mitigate ongoing blood clotting and improve perfusion quality, ensuring that the preservation solutions can effectively reach all parts of the brain, although they may not be as effective in the post-mortem setting once clots have already formed. Thrombolytics such as streptokinase or alteplase may be used to actively break down existing clots, potentially improving perfusion in cases where clot formation has already occurred [24]. However, their use must be carefully considered due to potential risks such as hemorrhage, especially in the context of compromised vascular integrity post-mortem. Neuroprotectants, like propofol, minocycline, s-methylisothiourea (SMT), and melatonin, can be employed to mitigate the effects of ischemia and protect the brain’s ultrastructure. Medications such as mannitol and decaglycerol can be administered to reduce cerebral edema, with the goal of enhancing perfusion quality. pH buffers such as tromethamine can be administered to mitigate acidosis. Post-cardiac arrest stabilization can also involve the use of vasopressors, such as vasopressin, to maintain adequate perfusion pressure during chest compressions or extracorporeal circulation and optimize the distribution of preservation solutions throughout the brain. Notably, the ideal goal of the most advanced stabilization procedures when implemented rapidly after cardiopulmonary legal death is to restore and maintain the brain in a functional, metabolizing, anesthetized state compatible with contemporary recovery prior to cryoprotectant perfusion [21,23]. 

While some stabilization medications are invaluable or ethically essential (e.g., anticoagulants, anesthetics, cardioplegic agents, pH buffers, pressors, and plasma volume expanders when needed), the value of others (e.g., free radical scavengers, anti-inflammatories, iNOS inhibitors, and PARP inhibitors) is based on the scientific literature, theory of ischemia-reperfusion injury, and experiments recovering mammals from long periods of warm ischemia without neurological deficit [10,25]. Medications and procedures that improve the perfusability of preservation solutions can be identified and validated by studying perfusion after simulated ischemia and stabilization. However, medications for protecting functional brain viability, particularly medications derived from study of whole mammal resuscitation and long-term recovery, might not observably improve perfusability or structural endpoints in micrographs. They are instead based on the principle that treating a post-ischemic brain in a manner believed compatible with successful contemporary resuscitation is the most conservative approach for post-ischemic brain preservation because damage that cannot be seen on micrographs today may still impact the success of biostasis.

Because ischemia-reperfusion injury is multifactorial, studying the brain resuscitation efficacy of individual agents in isolation is usually ineffective. Large-mammal resuscitation experiments are also difficult and expensive. Progress in mitigating cerebral ischemia-reperfusion injury in clinical medicine has therefore been slow. These same difficulties apply to the construction of post-ischemia medication protocols that seek to keep a brain contemporarily resuscitable before biostasis preservation. The design of such protocols depends upon literature surveys and resuscitation expert knowledge. The final arbiter for post-ischemic brain resuscitation is post-ischemic brain resuscitation and neurological assessment after long-term recovery.

Ischemic injury is not intrinsic to biostasis. If biostasis were to become an elective medical procedure, cardiopulmonary bypass would be surgically established. For example, in one proposed protocol, oxygenated blood, then cold oxygenated blood substitute, and finally cryoprotectants or other preservation solutions, would flow through the brain without interruption [26]. However, this is presently not legally possible.

Initial cooling techniques are another crucial component of the stabilization phase, as rapid cooling of the brain is essential for preserving its structure and minimizing ischemic damage. When stabilization is begun quickly after cardiac arrest by reestablishing blood circulation with external cardiopulmonary support, cooling has an especially important role for reducing metabolic demand of the brain so that the limited oxygenation provided by external chest compressions is adequate to support brain metabolism without additional hypoxic injury. Mild hypothermia during the early minutes of rapid cooling is also thought to be beneficial for brain recovery from ischemia-perfusion injury following warm ischemia.

Cooling is also important when it is not possible to promptly restore blood circulation. Cooling of the head and therefore the brain is the main technique used in aiding the preservation process when brains are donated to science [27]. Some brain banks measure the time it takes for the body to be refrigerated as the “refrigeration interval” [28]. There is extremely solid evidence that cooling the brain slows the process of brain cell decomposition during ischemia [29,30]. 

There are questions of how cooling should best be implemented in different settings. In this roadmap, we describe the use of various cooling methods, including external cooling with ice/water mixtures, ice packs or refrigeration, lavage techniques (colonic, peritoneal, nasopharyngeal), liquid ventilation, and cooling via the circulatory system. The most effective ones depend on restoration of blood circulation for effectiveness, either by chest compressions or extracorporeal circulation. This drives the need for medications to reduce reperfusion injury, and to establish parameters for the duration of ischemia after which prolonged reestablishment of blood circulation to accelerate cooling may be contraindicated by the severity of reperfusion injury. If circulatory arrest is required during surgery to establish extracorporeal circulation, then another important question is how much cooling should occur by chest compressions before circulation is stopped for the expected duration of the surgery before circulation is restored. The research goal here is to determine which methods or combinations thereof can most effectively and rapidly cool the brain in different contexts, thereby improving perfusion quality and neural preservation. Finally, the effects of oxygenation on the neural structure after different post-ischemia windows is another area for research, with the goal of determining whether this improves the quality of the stabilization process and minimizes damage to the brain [31].

## 4. Preservation Compounds

There is a need for further research in both the chemicals used for preservation as well the methods of delivering these compounds to the brain. A naïve view might be that the effective delivery of preservation compounds is the primary challenge, given that provably reversible cryopreservation has been achieved in cells [32], small tissue samples [33], small organisms [34], and even small mammalian organs [35,36,37,38,39]. However, scaling up reversible cryopreservation methods to the entire human brain would most likely require significant advancements in both the development of improved mixtures of preservation compounds and their effective delivery to the entire brain. Several factors highlight the complexity of this challenge. First, the role of the cooling rate in cryopreservation is important. Even though cooling organs as large as the human brain is possible without ice formation because of the low critical cooling rate of some contemporary cryoprotectant mixtures developed for organ cryopreservation [40], the time-dependent accumulation of toxic effects of cryoprotectants during cooling is a significant obstacle to reversible cryopreservation of large organs. Second, unlike other organs, the vascular system of the brain does not have capillary gap junctions that permit small cryoprotectant molecules to easily diffuse from blood vessels into the interstitial space. The brain instead possesses a blood–brain barrier (BBB) that, if not opened in some manner, requires that cryoprotectant molecules diffuse through vascular endothelial cells before reaching the parenchyma. This slows the addition of cryoprotectants to brain tissue and removes water from the interstitial space in larger volumes than cryoprotectants replace, causing mechanical distortion and elevated concentrations of endogenous proteins and toxic salts. 

The first class of preservation chemicals we cover in the roadmap are various interventions and additives that aim to enhance the quality of chemical delivery to the brain parenchyma, while also minimizing damage during the preservation process. The most important interventions in this group are those that open the BBB, either through chemical means, like sodium dodecyl sulfate [17,41,42,43,44], or using ultrasound techniques [45]. Overcoming the BBB is the primary challenge in ensuring that preservation agents effectively reach brain tissue, especially in more optimal conditions with low ischemia (because ischemia itself can also cause the BBB to open). For example, in cryopreservation approaches, an intact BBB causes cryoprotection outside vessels to depend excessively on endogenous solutes instead of less toxic cryoprotectants. This happens because cryoprotectants cross the BBB into the brain parenchyma more slowly than water leaves in the other direction in osmotic response to the cryoprotectant in vessels. Cryoprotectants must penetrate the BBB by passing through capillary endothelial cell membranes by passive diffusion, a process facilitated by hydrophobicity and a molecular mass less than 400 g/mol [46]. Conventional cell-penetrating cryoprotectants, such as DMSO, have a molecular mass less than 100 g/mol, which is helpful for diffusion, but have limited hydrophobicity. They therefore cross the BBB more slowly than water. The limited penetration that does occur is empirically sufficient for brain vitrification when combined with resulting brain dehydration [10,47], but this dehydration is undesirable because of mechanical distortion and toxic effects of concentrated salts. If the BBB is opened prior to cryoprotectant perfusion, techniques for cryopreservation are expected to function more similarly to those used in other organs lacking a ubiquitous selective vascular barrier, such as the kidney, where cryopreservation approaches have had significant success [35,38,39]. However, too much BBB disruption can cause excess fluid accumulation in the brain parenchyma, so this must be carefully balanced [48]. For aldehyde-stabilized cryopreservation, in animal experiments, opening of the BBB in conditions without ischemia leads to preservation of the brain ultrastructure [17]. Yet, there are still open questions regarding the best approaches for combining BBB modification techniques with fixation in cases with prior ischemic insult. Thus, modifications to the BBB should be front-and-center in biostasis research as a central problem to address for effective brain preservation in many circumstances. 

A second class of preservation compounds are those that aim to increase the flow of chemicals through the vascular system. This becomes relatively more important in suboptimal conditions for biostasis. One type of chemical of interest here is those that influence the osmotic properties of the perfusate, because a relatively higher osmolarity can be associated with improved perfusion [49]. Various chemicals can also be used to help dissolve blood clots, which can impede the perfusion of preservation solutions after periods of vascular stasis [50,51]. By promoting flow through the vascular system, these compounds aim to improve the extent to which preservation solutions can effectively distribute throughout the brain’s vascular network. However, because some of these chemicals may also act as detergents and cause damage to lipids and other structures in the brain, it will be important to balance the potential benefits of any given chemical in improving perfusability with their potential downsides. 

The success of cryopreservation largely depends on the ability of cryoprotectant mixtures to prevent ice crystal formation, minimize cellular damage, and maintain the structural integrity of the brain during the cooling and rewarming processes [52]. There is also a significant overlap between the development of cryoprotectants for brain cryopreservation and the field of organ cryopreservation for transplantation, which is rapidly growing and gaining higher levels of funding [36]. Advances in organ cryopreservation research already have and likely will continue to contribute to the development of cryoprotectant agents (CPAs) that could be adapted for use in biostasis. For example, the M22 solution used by the Alcor Life Extension Foundation was originally developed for kidney vitrification [53,54]. However, it is important to note that the unique challenges posed by the brain, such as its blood–brain barrier, complex structure rich in lipids, high metabolic demands, and sensitivity to ischemia, may require brain-specific cryoprotectant formulations. The economic incentives for developing brain-specific cryopreservation technologies for biostasis are currently limited compared to those for organ cryopreservation in transplantation. We use the term “cryopreservation technologies” because the problem is not just about the CPAs used—as the choice of solutes is so limited by nature—but rather about the whole mixture including non-CPA additives, as well as the methods of administration. 

In cryopreservation science, CPA molecules are classified as either penetrating or non-penetrating, depending on whether they are small enough to cross cell membranes to act intracellularly (typically <100 g/mol). One research topic is improved penetrating CPA mixtures with the goals of lowering the critical cooling and warming rates necessary to avoid ice formation, reducing toxicity, and enhancing perfusability. There is a need to better understand the efficacy of various existing CPA mixtures, such as the mixtures M22 and VM-1 [55,56]. Additionally, research can focus on experimental systems designed to screen new CPA candidates and candidate mixtures for their eventual potential for preserving the brain and other tissues. There is a need for experimental systems to enable the evaluation of cryoprotectant performance in a more realistic context, taking into account factors such as tissue heterogeneity and the challenges of perfusing a large, complex organ like the brain. The field would benefit from extensively querying the effects of potential novel cryoprotectant mixtures and other compounds with the goal of toxicity reduction.

In addition to the core penetrating cryoprotectants, there is also a need to query the efficacy of other non-penetrating CPAs that may enhance neural viability and structural preservation. These include biologically inspired compounds like ice blockers, which can further inhibit ice crystal formation, necessarily accompanied by research on how to get large molecules to interstitial and intracellular locations. 

While cold tolerance in nature has inspired development of some non-penetrating CPAs like ice blockers as analogs of antifreeze proteins, natural freeze tolerance has been less useful as a guide for problems of solid-state cryopreservation. Animals that survive freezing in nature do not actually freeze to a solid state compatible with long-term preservation. Freeze-avoiding animals retain the liquid state of all their body fluids, and freeze-tolerant animals like certain frogs retain the liquid state of their cells by allowing ice to form outside cells and outside organs that remain liquid inside at natural temperatures [57]. With the possible exception of some insects [58], the concentrations of CPAs in cold-tolerant animals are too low to allow survival of cooling to the glass transition temperature necessary for solid-state preservation. The development of CPA mixtures permitting reversible solid-state mammalian organ preservation has required diverging from the natural world, with successes relying on small penetrating CPAs not used in nature, including DMSO, ethylene glycol, and formamide [35,38]. 

For fixatives, it is not as clear that any fundamental advances are needed. Formaldehyde has been used with substantial efficacy in preserving the brain’s structures since the 1890s, while glutaraldehyde came into widespread use in this area in the 1960s [59,60]. These aldehyde-based fixatives have proven to be effective in preserving neural structures for microscopic studies, and they have long been gold standards for histological and electron microscopy applications [61]. However, there is still room for optimization in the use of these fixatives for biostasis. One area of focus is the optimization of aldehyde fixation protocols for preserving biomolecular properties. While aldehydes are effective at preserving morphology, they also cause extensive crosslinking of proteins and other biomolecules, which can hinder downstream molecular analyses. Researchers are investigating ways to measure the extent of biomolecular property restoration via crosslinking reversal with today’s technology. For example, catalysts have been shown to help remove formaldehyde crosslinks, enabling better molecular profiling of fixed tissues [62]. Further research in the reversal of aldehyde crosslinks may help to elucidate whether and how future technologic methods may be able to reverse them, while also being useful for contemporary research applications. Additionally, studies could be conducted to compare the effectiveness of different aldehyde formulations, such as methanol-stabilized formaldehyde versus formaldehyde prepared from paraformaldehyde, in preserving neural structures. The extant literature is still not conclusive on whether the small concentration of methanol in commercial formalin may lead to worse histological outcomes [63,64,65]. 

Another area of interest for fixation compounds is the development of chemicals that can accelerate immersion fixation, enabling better preservation of deep neural structures. Some literature suggests that certain compounds, such as dimethyl sulfoxide (DMSO), can enhance the penetration of fixatives into tissue, potentially improving the preservation of deeper brain regions [66]. Non-aldehyde crosslinkers, such as 3,3′-dithiobispropionimidate and dithiobis(succinimidyl propionate) (DSP), could also be explored as alternatives to traditional aldehyde fixatives. Some non-aldehyde crosslinkers have the advantage of being cleavable with contemporary technology, allowing for the contemporary reversal of crosslinking and potentially better preservation of biomolecular properties. For example, studies have shown that preservation with DSP can lead to improvements in biomolecular profiling compared to aldehyde fixation [67]. However, non-aldehyde crosslinkers lack the extensive evidence base for structural preservation, and their relative efficacy is questionable, so they would likely require substantial research and optimization prior to widespread use. In addition to optimizing fixation protocols, there could be more research into preservative compounds to maintain the morphology and biomolecular properties of the brain during long-term preservation in fluid. The long-term stability of lipids is a particular question that requires research and may depend on the temperature of storage, which could affect the biophysical state of lipid molecules [68]. Certain chemicals such as glycerol, ethylene glycol, and sucrose have previously been used to enhance the long-term stability of fixed tissues. Testing tissue that has already been preserved in fluid for decades, as well as conducting accelerated aging experiments, could provide valuable insights into the effectiveness of different preservatives in maintaining neural structures over time. Taken together, while aldehyde fixatives have a long history of success in preserving brain morphology, there is still room for improvement in their use for biostasis. 

## 5. Preservation Procedures

Just as it is critical to research improvements in preservation compounds, the importance of developing better delivery methods cannot be understated. Even the most advanced preservation compounds will be ineffective if they cannot be reliably and uniformly distributed throughout the brain, ideally through its vascular network. This is where research into delivery techniques, such as optimizing perfusion parameters, modifying the blood–brain barrier, and using novel delivery systems, becomes crucial. 

### 5.1. Surgical and Cannulation Technique

One of the key aspects of preservation procedures is the surgical and cannulation technique used to deliver the preservation compounds to the brain. Notably, this is distinct from surgical and cannulation techniques that might be used in stabilization. For example, femoral cannulation has been primarily used in biostasis in the context of stabilization. As a definitive preservation technique, it does not have any clear advantages in terms of perfusing the brain, other than ease of surgical access.

Several approaches have been employed, each with its own advantages and challenges (Table 3). Transcardial perfusion, which involves cannulating the heart to deliver the preservation solution into the brain’s vasculature, has been used in the past for brain preservation and is commonly employed in animal experiments [69,70]. Another approach is the direct aortic cannulation route, which has been used by Alcor and Tomorrow Bio for whole-body cases [71,72]. In both methods, the descending aorta can potentially be clamped, thus minimizing perfusion to areas of the body other than the brain and saving the cost of purchasing chemicals, although this can be technically challenging and is not always desired. Another approach is in situ carotid cannulation, which has been used by the Cryonics Institute and with some frequency for perfusion fixation in brain banking [73,74,75]. For example, in situ carotid perfusion fixation was found in one study to lead to high-quality ultrastructural-level preservation in donated human brains [76]. However, this method would not perfuse the portions of the brain supplied by the vertebral arteries, unless there is an intact circle of Willis, which may not be the case in a relevant portion of the population, due to variants and/or age-related changes in the vasculature. Even with an intact circle of Willis, vertebral arteries may need to be ligated to avoid loss of arterial pressure from retrograde flow out of the open arteries [77]. 

For individuals choosing cephalon preservation, Alcor has employed a cephalon-isolated, 2- or 4-vessel cannulation route [78]. Similar methods have also been used in the brain banking context [79,80,81]. This method involves isolating the cephalon from the rest of the body and accessing the major arteries supplying the brain. Theoretically, this allows cannulation of both carotid arteries and both vertebral arteries. However, it can be technically difficult to cannulate the vertebral arteries, which can be quite small, so this technique may take more time, or one of the vertebral arteries may not be able to be cannulated, especially if the circle of Willis is not patent [47]. The extent of perfusion pressure that will be achieved in the posterior circulation of the brain when the carotids are cannulated and the vertebrals are clamped will depend on the degree of perfusion impairment present and appears to be an open research question. Ex situ cerebral vessel cannulation, the most commonly used perfusion method in brain banking, involves removing the brain from the skull and directly cannulating its major vessels [82]. This method of perfusion fixation has been reported to increase preservation quality compared to immersion fixation in brain banking [83,84]. It allows for direct visualization of the progression of perfusion. However, it relies on the effective extraction of the unfixed brain with sufficiently intact vessels to perfuse, making it challenging to avoid factors that will lead to perfusion impairment, such as compression of vessels or air bubbles. In addition to these four approaches, there have also been others used in cryopreservation cases, such as a subclavian approach that has been used by the Cryonics Institute [85]. 

**Table 3:** Upsides and downsides of different surgical and cannulation techniques for perfusion.

Approach	Upsides	Downsides
Transcardial perfusion or direct aortic cannulation (Used by Tomorrow Bio and Alcor for whole-body cases)	Transcardial perfusion is commonly used in animal experiments Allows perfusion of all four brain vessels with one cannula	Difficult to isolate perfusion to the brain, so theoretically, perfusate may go elsewhere in the setting of differential perfusion impairment of the brain, requiring further study Technically challenging to clamp descending aorta, but otherwise perfusate will go to the whole body, which may not be desired
In situ carotid cannulation (Used by Cryonics Institute and infrequently in brain banking)	Relatively straightforward technical procedure Shown to lead to high-quality ultrastructural-level preservation in human brains [76] Perfusion to the face can be prevented by clamping the external carotid [73], but this takes time and is more technically challenging	In most cases only leads to perfusion in the internal carotid distribution, which supplies just 88% of the cerebrum by volume [86] Possible shunting of perfusate away from the brain via backflow through the vertebral artery [77]
Cephalon isolation, carotid cannulation with or without vertebral cannulation (Used by Alcor for cephalon-only preservation cases)	Could measure distinct perfusion flow rates or pressures to each vessel Decreases cerebral venous pressure because both jugular veins can freely drain [23]	Technically challenging and time-consuming to isolate and cannulate the vertebral arteries Sometimes vertebral cannulation cannot be performed, but in this case, the vertebrals can still be clamped to prevent shunting of perfusate
Ex situ cerebral vessel cannulation (When perfusion is used in brain banking, which is relatively uncommon, this is the primary method)	Reported to increase preservation quality compared to immersion fixation [83] Allows direct visualization of perfusion progression Requires only access to the brain	Relies on effective extraction of the unfixed brain with intact vessels Difficult to avoid factors leading to perfusion impairment, such as air bubbles, traction injury to vessels, or compression of vessels

In addition to the perfusion route, there are other aspects of the perfusion procedure that require optimization. Optimizing the range of perfusion pressure is crucial for ensuring uniform distribution of preservation compounds throughout the brain. In the brain banking context, it has been reported that lower pressures run the risk of incomplete perfusion, potentially leading to poor tissue preservation and histological artifacts [87]. Higher pressures, on the other hand, run the risk of causing ruptures in delicate cerebral vessels, disrupting the blood–brain barrier, and increasing non-physiologic collateral circulation that might bypass the brain [73,88]. One study in mice using normothermic perfusion found that perfusion pressures below the physiological systolic blood pressure resulted in the collapse of parenchymal vessels, formation of microvasospasms, and microclots, while pressures above the physiological systolic blood pressure dilated cerebral vessels, induced microvasospasms, and disrupted the blood–brain barrier [89]. The study concluded that a perfusion pressure between 125 and 150 mmHg is optimal for preserving both the cerebral vasculature and neuronal structures in mice. However, the situation for hypothermic brain perfusion is quite different, as blood vessels respond to perfusion differently at these temperatures, and the optimal perfusion pressure range is likely to be substantially lower. Overly high perfusion pressures can lead to brain swelling and herniation. Alcor recommends that perfusion pressure monitored in the arterial line should not exceed 100 mmHg during cryoprotectant perfusion [23]. 

There is also a question of how to prevent air microemboli and how much this helps preservation quality. It makes basic biophysical sense that air microemboli could become trapped in vessels and prevent subsequent flow. In cardiac surgery, which is relevant to biostasis as it often involves the perfusion of the brain with an external fluid, there is mixed evidence on whether air microemboli are associated with worsened cognitive outcomes post-surgery [90]. The extent and type of air microemboli removal that is of practical use in biostasis is uncertain and may depend on the surgical route used. Finally, there is a need to study how agonal factors, ischemic interval, and any particular disease state that the person may have affect the best way to perform perfusion. For example, it is still poorly mapped how perfusion quality changes during the interval of global cerebral ischemia prior to the initiation of the procedure, especially in human brains [87]. Factors such as the duration of ischemia, the presence of pre-existing vascular pathologies (e.g., atherosclerosis), and the individual’s age and medical history may all influence the optimal perfusion parameters and the overall success of the preservation procedure. Future research should aim to systematically investigate these factors and develop evidence-based guidelines for tailoring perfusion protocols to individual cases. 

### 5.2. Burr Hole Creation and Brain Extraction

Burr hole creation is a useful step in some biostasis procedures that allows for the visual monitoring of the brain’s response to the perfusion process [23]. For example, two small holes can be drilled into the skull using a standard neurosurgical tool, such as a Codman perforator. These burr holes provide a window into the brain’s condition, enabling the biostasis team to assess the extent of ischemic injury and the effectiveness of the perfusion. If the brain exhibits substantial swelling, it may indicate a disruption of the blood–brain barrier, damage to blood vessels, a need to reduce perfusion pressure, or inadequate osmotic concentration of the perfusate [49]. If profound cerebral edema or elevated intracranial pressure is observed through the burr holes, it may be necessary to terminate perfusion. Additional research may be justified on the best location(s) to place the burr hole(s) and how to best interpret and use the resulting tissue visualization that they allow. 

Brain extraction is another important aspect of some biostasis procedures that can serve multiple purposes, such as determining tissue preservation quality in research studies, enabling immersion fixation in chemical preservation approaches, or facilitating ex situ perfusion. The process of brain extraction involves several steps: (1) soft tissue removal, (2) skull removal, (3) dura removal, (4) severing of the connections (nerves, blood vessels, spinal cord), and (5) extraction of the brain from the skull. There are multiple ways to perform brain extraction, each with costs and benefits that are not yet well established from a biostasis perspective [91,92,93]. In large-animal brains, an alternative approach for immersion fixation is to remove the skullcap while leaving the brain inside the skull until it is adequately fixed, which may help prevent damage during the extraction process [94]. This technique could potentially be adapted for use in human biostasis cases, particularly when immersion fixation and cryoprotection is indicated instead of perfusion because of compromised circulation due to ischemic injury or other factors. Overall, further research is needed to validate the effectiveness of different brain removal approaches in human brains and to develop standardized protocols that optimize preservation quality.

### 5.3. Cryopreservation-Specific Procedural Considerations

For pure cryopreservation approaches, rapid cooling techniques are often a critical aspect of the procedure, as they aim to minimize the formation of ice crystals and reduce cellular damage during the cooling process. It is less critical for cryopreservation following aldehyde fixation as this method allows the use of higher concentrations of cryoprotectants. Developing effective procedures for fast cooling to cryogenic temperatures in large tissue volumes, such as the human brain, is a significant challenge in cryobiology that requires further research. Studies could focus on establishing new cooling procedures and evaluating their effects on neural viability and structure. This may involve investigating the use of novel cooling agents, optimizing cooling rates, and assessing the impact of different cooling techniques on various regions of the brain. Cooling can be accelerated by introducing, under pressure, cold gas or liquids into the vasculature, such as silicones or fluorocarbons that remain liquid at very cold temperatures [95,96,97,98,99]. Options for the gas used include oxygen, air, nitrogen, and helium [100]. Cooling using gas is not yet well-established to allow for brain preservation, even in animal models. However, if the associated biological challenges can be solved, it has potential to vastly enhance the rate of cooling, at least in ideal cases where perfusion quality is very high. Establishing standardized research protocols for cooling with gas and investigating its effects on neural viability and structure would be a useful step towards this research goal. 

As extensively discussed above, effective perfusion of cryoprotectants is a key consideration in cryopreservation procedures. An additional research area towards this goal is optimizing the basic parameters of perfusion procedures, such as using different forms of pulsatile flow and cryoprotectant concentration ramp-up curves. The best parameters for different perfusion techniques and their impact on neural viability and structure will be specific to each procedure, underscoring the need for both basic research into the best overall procedural approaches and applied research into how to practically implement them. Additionally, fracturing is a common issue encountered during the cryopreservation of large organs, such as the human brain. Optimizing cooling speed to reduce thermal stress and minimize fracturing is another important goal for future research. 

Finally, unprotected cryopreservation or “straight freeze,” which involves cooling the brain without the use of cryoprotectants, is a topic that warrants further investigation. While this approach results in significant ice formation and cellular damage [101], quantifying the extent of this damage and its effects on neural structure can provide valuable insights into the limits of cryopreservation techniques. It remains unclear exactly how much damage to the connectome occurs when using different cooling and rewarming methods and how this might depend on patient characteristics such as the amount of ischemic damage they have experienced. By studying the outcomes of unprotected cryopreservation on brain structure, researchers can also better understand the role of cryoprotectants and inform the development of more effective preservation strategies that do employ them.

### 5.4. Fixation-Specific Procedural Considerations

For chemical fixation, there are several specific procedural considerations that warrant further research. Immersion fixation, which involves submerging the brain in a fixative solution, is a widely used method in brain banking and neuroscience research. However, the effectiveness of immersion fixation in preserving both superficial and deep neural structures needs to be thoroughly benchmarked. Studies could compare the quality of preservation at different depths within the brain and assess the impact of factors such as temperature, fixative composition, and immersion duration. Additionally, researchers could investigate whether more invasive methods, such as needle injection of fixatives into the brain parenchyma, targeted cuts to enhance fixative penetration, or the “pseudoperfusion” of fixatives through the ventricular system [102], can improve overall preservation quality during immersion fixation. Perfusion fixation, which involves pumping fixative solutions through the brain’s vascular network, is another important area of research. One key question is the necessity of using washout solutions prior to perfusing fixative-containing solutions. The literature is conflicting on whether washout solutions actually improve the quality of perfusion fixation, with some sources suggesting that they may help remove blood clots and improve fixative penetration, while others suggest that they are not necessary, leading to a delay in fixation and an increase in procedural complexity [87,103]. When formaldehyde alone is used, fixation is a relatively slow process taking at least minutes to hours to be completed, so there is less of a concern for perfused fixatives to cause blood clots to be trapped in situ immediately, although this is another aspect of the procedure that is worthy of research. Systematically comparing the quality of perfusion fixation with and without different washout solutions in animals with large brains and simulated ischemia could help resolve this debate and optimize fixation protocols. 

Post-fixation cryoprotectant delivery is another crucial consideration for chemical fixation in the context of biostasis if storage is to be done at sub-zero temperatures. Storage is ideally at temperatures cold enough to result in vitrification, which is the achievement of a solid state below the glass transition temperature (Tg), so that translational motion of all biomolecules is practically arrested. Cryoprotectants can be delivered to the fixed brain through either perfusion or immersion, and the choice of delivery method may depend on factors such as the patient’s condition, the available resources, and the desired outcome. Perfusion-based delivery of cryoprotectants is used in aldehyde-stabilized cryopreservation and has the key advantage of allowing for more rapid preservation, leading to less diffusion of unfixed biomolecules such as small molecules [17]. However, it relies on high-quality perfusion, which may not always be possible. The use of perfusion fixation followed by cryoprotectant perfusion warrants significant further study in conditions that mimic the challenges often encountered in biostasis, such as the presence of ischemic or agonal damage. Researchers could compare different perfusion methods and assess their impact on neural structure preservation. Similarly, immersion-based delivery of cryoprotectants could be optimized by investigating different cryoprotectant immersion ramp-up protocols and their effects on neural structure. By systematically evaluating these post-fixation cryoprotectant delivery methods, further research could develop evidence-based protocols that maximize the potential for successful long-term preservation of chemically fixed brains.

## 6. Measuring Preservation Quality

### 6.1. Structural Preservation of the Brain

When discussing the “structure” of the brain, we refer to both the biomolecules that serve as the building blocks of brain structures and the higher-level features that they compose, which can be visualized under the microscope. One of the key challenges for evaluating the appearance of cells and other microscopic structures is that we do not know with certainty what structures are required for brains to produce and maintain memories, emotions, and other aspects of our functioning. However, extant evidence and our most widely accepted models suggest that the action is occurring at the cellular and subcellular levels, such as neurons, dendrites, synapses, astrocytes, and myelination. Researchers could establish light microscopy metrics, such as the expected cellular morphology across different brain regions, to evaluate preservation at a cellular level in a more scalable manner. These metrics could include assessments of neuronal shape and the presence of intact cell membranes. At the ultrastructural level, metrics such as the traceability of neuronal processes on electron microscopy or expansion microscopy could be further developed to assess the preservation of fine cellular details such as axons, dendrites, and synapses [104,105]. The statistics of neuronal connectivity, both local and long-range, appears to be a particularly important parameter to measure whether it is intact after the preservation procedure. Long-range connectivity is currently difficult to measure, but future advances in microscopy may allow this. Biomolecular annotation metrics are another important aspect of measuring preservation quality. These metrics aim to determine whether the distributions of various biomolecules, such as proteins, lipids, and nucleic acids, are preserved in their native states. Techniques such as expansion microscopy, spatial proteomics, spatial transcriptomics, immunohistochemistry, and immunoelectron microscopy could be used to visualize and quantify the preservation of biomolecular distributions [106]. The inferability of structural appearance could also potentially be investigated using contemporary machine learning techniques. By training machine learning models on well-preserved brain samples and testing their performance on damaged or partially preserved samples, researchers could establish the extent to which the initial structural information can still be inferred in the setting of suboptimal preservation. 

While we have outlined several potential metrics for evaluating brain tissue preservation, it is important to note that standardized, widely accepted structural preservation metrics specifically for biostasis do not yet exist. Exactly what preservation of the connectome means can be quite unclear. For example, does this require preservation of structures in the exact manner as is seen in laboratory animals under ideal conditions? This seems like too stringent of a threshold, considering that there are some structural features that are altered in the first few minutes after clinical death, within the window that is known to be reversible by contemporary cardiopulmonary resuscitation [30]. However, if the benchmark is not the same preservation quality that is seen under ideal laboratory conditions, it is unclear exactly where the threshold for good enough should be drawn. Most likely, it is not binary. As a result, improved, nuanced structural preservation metrics will need to be developed and validated, while attempting to capture the most likely structures involved in key cognitive functions such as long-term memory. As better structural preservation metrics are established, it may be easier to drive progress towards improved methods for preserving the structure of the brain.

### 6.2. Cryopreservation-Specific Metrics

Intra-preservation quality control measures are important for assessing the quality of cryopreservation while the process is ongoing. One such metric is the refractive index of venous effluent perfusate during cryoprotectant perfusion, which can provide information about the state of osmotic equilibration of cryoprotectants in the brain. For intra-preservation quality metrics, there is a critical need to investigate the relationship between the metric and the ultimate quality of neural preservation, as assessed by more definitive metrics such as cellular viability and structural integrity. This will help us to evaluate their value in guiding the procedure. 

One promising set of post-preservation quality control measures is CT-based metrics [107,108], which could provide non-invasive, high-resolution images of the brain both during cryoprotectant perfusion and after completion of cryopreservation. CT scanning has already been very useful for quantifying the apparent cryoprotectant concentration and extent of ice avoidance after cryopreservation of the brain [109,110,111]. A research goal here is in establishing standardized CT-based preservation metrics, such as measures of tissue density, contrast, and uniformity. It is also important to correlate CT-based imaging metrics with more direct assessments of cellular and structural preservation to ensure that they are adequate proxies of tissue quality. 

Another important post-preservation quality metric is the assessment of structural preservation after rewarming, which refers to the process of rewarming a vitrified brain to its non-vitrified state. Different types of processing during and after warming can reveal the structural state of tissue during different phases of the preservation and recovery process. Freeze substitution, i.e., the replacement of ice with solvent and fixative at deep sub-zero temperature, will reveal the structural state of tissue in the cryopreserved state before ice melts and rehydrates cells. This is of particular utility for brains cryopreserved by freezing. For cryopreservation by vitrification, the structure of the brain in the cryopreserved state can be studied by chemically fixing the brain while still loaded with cryoprotectant either before cooling or after warming from vitrification. This is possible because cooling to the vitrified state, or warming from it, does not change the morphology of the tissue if ice formation is avoided. This fact conveniently allows vitrification protocols to be evaluated during development by simply loading with cryoprotectant without actually vitrifying, provided that ice avoidance during cooling and warming is later verified.

Even though vitrification avoids structural damage from ice, brains that are fully-loaded with a vitrification solution may be morphologically altered by osmotic shrinkage of cells or the whole organ, making it difficult to interpret to assess connectome preservation in micrographs [25]. For this reason, it is useful to do partial or complete perfusion unloading of cryoprotectant from brains before fixation for structural assessment. However, this can add structural damage from osmotic effects during the unloading process that is not present in the preserved, fully loaded state. 

Whenever chemical fixation of a brain that still contains cryoprotectant is performed, it is critically important that the fixative solution contains the same or osmotically similar concentration of cryoprotectant so as to not add further osmotic damage as an artifact of fixation. After fixation, the cryoprotectant can be removed by gradual reduction in its concentration in the perfused fixation solution, or by serial dilution of the cryoprotectant by placing extracted fixed tissue pieces into vials containing fixative with progressively lower cryoprotectant concentration until only ordinary fixative solution remains. Further tissue processing may then proceed in a conventional manner.

Establishing reliable post-rewarming structural preservation metrics, including measures of cellular morphology, is also essential for accurately assessing the quality of cryopreservation. In particular, it remains uncertain whether vitrification protocols not employing prior chemical fixation meet the criteria of preserving the structural connectome across the brain [104], as aldehyde-stabilized cryopreservation has achieved [112] by preserving brain structure in electron micrographs that is indistinguishable from that of non-cryopreserved controls [17]. 

In addition to structural preservation, post-rewarming cellular viability and functional metrics are also important for evaluating cryopreservation quality. For whole brains, achievement of these preservation milestones will likely be largely at a later stage than that of structural preservation metrics. An initial research goal is to establish standardized assays for measuring cellular viability in rewarmed brain tissue, such as tests for metabolic activity, membrane integrity, and apoptosis. By comparing the cellular viability of rewarmed brain tissue to that of fresh, non-cryopreserved tissue, researchers could then gain valuable insights into the effectiveness of different cryopreservation protocols, as was done for VM3 [113], a close relative the M22 solution that has been used for whole-brain vitrification [10]. Post-rewarming electrophysiological activity is also useful to study, and particularly in studies showing functional preservation of electrophysiological responses corresponding with memory encoding. Researchers could establish post-rewarming neural activity metrics by studying unsynchronized and synchronized electrophysiological activity patterns in isolated brain preparations or brain slices [114,115]. One study found that electrophysiological responses can be retained after vitrification of mouse corticohippocampal slices, albeit with reduced field excitatory postsynaptic potential amplitude [116]. Additionally, one study already demonstrated a retained ability to form a long-term potentiation (LTP) response in vitrified and rewarmed hippocampal slices [117]. By comparing these electrophysiologic activity patterns to those observed in fresh, non-cryopreserved tissue, researchers could gain valuable insights into the extent to which cryopreservation can preserve the functional integrity of the brain. 

With any interpretation of brain slice experiments, it is critical to recognize that the structure and function of brain slices after diffusion loading and unloading of cryoprotectants can differ from the structure and function of brain tissue within an intact brain after perfusion loading and unloading of cryoprotectants. Unlike brain tissue slices, or other organs with capillary gap junctions, the movement of water and cryoprotectants inside a brain is modulated by the presence of the blood–brain barrier that stands between perfused solutions and brain tissue beyond. While this has not been found to be an obstacle to brain vitrification in terms of ice avoidance—because water moves relatively rapidly to osmotically equalize water activity and the freezing tendency of intravascular and extravascular compartments during cryoprotectant perfusion—the chemical composition of the intravascular versus extravascular compartments can be quite different during whole-brain vitrification. Functionally successful slice cryopreservation does not automatically translate to successful organ cryopreservation, especially for the brain. In particular, avoiding osmotic damage during complete unloading of cryoprotectant from an intact brain is much more tedious and difficult than it is for a brain slice. 

The ultimate assay for assessing reversibility of a brain cryopreservation protocol after rewarming, and after perfusion unloading of cryoprotectant, is reperfusion of the whole brain with warm, oxygenated blood [114]. Electroencephalographic, metabolic, and, in some models, behavioral assays can then be performed to assess the function of the brain as an integrated organ. Ethical considerations are paramount in such assays, including anesthesia, analgesia, and sedation to the maximum extent possible for measurements being performed. The ethical status of a resuscitated brain is the same as that of a whole animal or person.

Demonstrably reversible preservation of whole brains is a necessary platform upon which future long-term suspended animation of whole mammals or people must be built. Efforts to reversibly cryopreserve whole large animals are not credible before technology at least exists to reversibly cryopreserve discrete tissues and organs, most especially the brain.

## 7. Long-Term Preservation

The long-term preservation of the brains of patients is a critical aspect of biostasis, as it aims to maintain the structural and potentially functional integrity of the brain over extended periods, possibly centuries or longer. The storage conditions and methods employed can have a significant impact on the quality of preservation and the potential for future revival.

For cryopreserved brains, the temperature of storage is a key factor to consider. More research is needed to investigate the effects of different storage temperatures on macroscopic and microscopic brain structure, cells, and biomolecules. A critical factor is the glass transition temperature (Tg) of the cryoprotectant solution used, which determines the temperature range at which the brain can be stored without the risk of ice crystal formation or chemical change because the translation of molecules is practically arrested. For example, current cryopreservation protocols typically involve storage in liquid nitrogen at −196 °C, which is well below the Tg of all cryoprotectant solutions. While this method is inexpensive, sustainable, and relatively easy to maintain, it may not provide the optimal conditions for long-term brain preservation. 

Intermediate temperature storage (ITS), typically between −140 °C and −130 °C, has been proposed as a potential alternative to liquid nitrogen storage [118,119]. The rationale behind ITS is that maintaining a temperature closer to the glass transition point of the cryoprotectant solution could minimize thermal stress and fracturing of the preserved tissue. When a vitrified brain is cooled below its Tg, the faster cooling rate of the warmer interior compared to the outer shell can cause differential thermal contraction, leading to fractures and structural damage [118,120]. This type of stress can be reduced by slow cooling or annealing near Tg. However, permanent stress due to different thermal expansion coefficients of different parts of vitrified specimens, such as the brain and the cranium in contact with it, cannot be relieved by time. Such stresses accumulate with greater temperature descent below Tg, unavoidably increasing fracture risk with further cooling. By storing the brain at a temperature just below its Tg, this form of damage could potentially be mitigated. 

However, implementing ITS systems for long-term brain preservation presents several challenges and research questions. First, ITS dewars typically have a smaller capacity and require more frequent refilling compared to immersion dewars, which increases the risk of temperature fluctuations and potential damage to the preserved tissue [118]. Second, ITS systems consume more liquid nitrogen and require more complex maintenance, resulting in higher costs compared to traditional liquid nitrogen storage. Third, there is a need for more empirical studies on the effects of different storage temperatures on cell structure, viability, and thermal stress fractures. Fourth, it is unclear how much damage these fractures do to the connectome compared to other forms of damage during preservation. Finally, storage too close to Tg can also cause nanoscale ice nucleation [120], which might make ice avoidance during future warming more difficult even if the nucleation itself is not expected to be significant biologically. The time dependence of ice nucleation in vitrification solutions as a function of temperature near Tg is understudied. Whether nanoscale heterogeneous ice nucleation can continue for centuries, or whether it plateaus after a limited population of heterogeneous nucleating centers is exhausted, is unknown.

Aldehyde-stabilized cryopreservation (ASC) has a particular advantage for fracture avoidance [17]. Fracturing in tissue preserved by ASC might be completely avoidable by storing at temperatures several degrees above Tg of the cryoprotectant solution used. Slow diffusion at viscosities ten orders of magnitude greater than water [120,121] is unlikely to change the preservation state of the chemically fixed tissue because enzymes are inhibited by protein crosslinking. It might also be argued that since fixation itself eliminates biological viability, slow loss of biological viability that might otherwise occur during non-solid storage is not a concern because there is no functional biological viability left to be lost. Furthermore, since the non-viable state of tissue preserved by ASC somewhat addresses concern about cryoprotectant toxicity, the cryoprotectant solution can be made so concentrated that ice nucleation is practically impossible as long as this high concentration is uniform throughout the tissue.

In addition to more study on storage temperature, the development of long-term quality metrics for cryopreserved brains is also essential for monitoring preservation quality over time. These metrics could include assessments of any structural changes visible on neuroimaging, such as ice crystal formation or fractures, as well as measures of cellular viability and functionality in research cases where samples can be rewarmed and evaluated. By evaluating the effects of storage conditions on these metrics at different time points, research could identify potential issues and develop strategies to mitigate them.

Long-term fluid preservation is another option for the long-term preservation of chemically fixed brains. In this approach, fixed brains are stored in a preservative solution at above-freezing temperatures [122]. In addition to the aforementioned need for improved chemical compounds to aid in fluid preservation, there is also a need for more research on other factors, such as the temperature, storage container, and other environmental conditions, that can affect this process. While fluid preservation is a cost-effective method that has the potential to allow broad access to biostasis, it remains far from guaranteed that it will be sufficient for preservation of biomolecules and cellular structures on the timescale of centuries, and therefore requires significantly more research. Accelerated aging experiments, in which fixed brains are exposed to elevated temperatures or other stressors, could also help predict the long-term stability of fluid-preserved brains and identify potential issues related to biomolecular degradation or structural changes.

## 8. Potential Restoration and Recovery

### 8.1. Provably Reversible Preservation Procedures

For provably reversible preservation procedures, the only plausible methods that could be developed anywhere in the foreseeable future are based on cryopreservation, as chemical fixation causes too many alterations and would require the development of much more advanced revival technology, such as advanced molecular nanotechnology or potentially even whole-brain emulation. A key challenge in reversible cryopreservation is the rewarming process, as improper rewarming can lead to ice crystal formation, ice recrystallization (the time-dependent formation of a smaller number of larger ice crystals from a larger number of smaller crystals), accumulation of cryoprotectant toxicity, and other damaging effects that compromise the function and viability of the preserved tissue. Several rewarming methods are under investigation, including dielectric warming, which uses electric fields to uniformly heat the tissue [39]; nano-rewarming, which employs magnetic fields to heat nanoparticles within the vascular system [38]; and ultrasound rewarming, which uses high-frequency sound waves to rewarm cryopreserved tissue [123]. Additionally, the development of novel rewarming technologies, perhaps leveraging recent advances in fields such as nanomedicine or bioengineering, could revolutionize reversible cryopreservation. In all cases, the procedures must be rigorously tested to demonstrate the preservation of the fine tissue structure, restoration of normal biological functioning, and recovery of the original information content that defines the individual. Proving the consistent reversibility of a given cryopreservation protocol is essential for instilling confidence that the procedure could potentially enable future revival. While reversible long-term cryopreservation of a human brain, or the brain of any other mammal, has not yet been achieved, there have been successes in the cryopreservation of mammalian organs [35,36,37,38,39], brain slices [113,117,124], and nematodes such as *C. elegans* [34]. Advances in rewarming technology, combined with improved cryoprotectants and vitrification cocktails, may eventually allow demonstrably reversible cryopreservation of whole mammalian brains to become a reliable method of biostasis for humans. 

### 8.2. Molecular Nanotechnology-Based Approaches

Another potential future technology that could be leveraged for revival from biostasis is molecular nanotechnology—the concept of engineering complex structures and machines with atomic precision [1,125,126,127]. This could enable repairing preserved tissues in a targeted way, or even reconstructing an entire organism from detailed scans. One proposed approach is to employ vast numbers of medical nanorobots capable of operating at the cellular and molecular scales [128]. These microscopic machines could be designed to perform specific tasks within the body. In the context of biostasis, specialized nanorobots could be used to carry out repairs on preserved tissue in a stepwise fashion. First, nanorobots could be introduced into the circulatory system to map out the tissue structure and assess damage [128]. Next, debris-clearing nanorobots could remove preservative chemicals, any ice crystals, any crosslinks from fixatives, and damaged cell components. Structural repair nanorobots would then stabilize membranes and reduce fractures. Finally, specialized nanorobots could precisely edit the genome and proteome of each cell, correcting any damage and restoring normal function [128]. Biological approaches to engineered molecular and cell repair are also theoretically possible [129]. 

For cases with more extensive damage precluding repair in situ—or if in situ repair never becomes possible—another potential option enabled by molecular nanotechnology would be to destructively scan the preserved brain at the molecular level and use that information to construct an atomically precise replica [128]. This could be done using massive arrays of mechanosynthetic tools to physically assemble the new brain, atom-by-atom, according to the scanned data. The scanning process could use advanced atomic force microscopy or related techniques to map out the position and type of every atom in the preserved brain. This incredibly detailed structural data would then be fed into the “nanofactory” responsible for constructing the replica brain. While the exact mechanisms for such advanced mechanosynthesis remain an area of high uncertainty, if it ever becomes possible, proposals generally involve using precisely controlled tips to remove hydrogen atoms or other molecular fragments from a surface, and then deposit the desired atoms or molecules in their place. By repeating this pick-and-place process many, many trillions of times, complex structures could be built up with atomic precision. The energy and control required at this scale would be immense, almost beyond current comprehension, but theoretical analyses suggest that it would not break any known laws of physics. While many of these concepts remain fantastical, ongoing research in fields like scanning probe microscopy, supramolecular chemistry, and biotechnology are bringing us closer to engineering complex systems at the molecular scale.

### 8.3. Whole-Brain Emulation

An emulator is a system that replicates the same internal causal processes as the original system, following all the rules and constraints of the emulated system, aiming to match the behavior of the original and its relevant components in every way [130]. In contrast, a simulation is a model that mimics an outward result—i.e., what output a system produces given some inputs—not necessarily capturing all the internal rules, meaning that the system can be treated as a “black box.” In the context of brain preservation, there is a long-standing idea that an emulation can serve as a form of potential revival, whereas a simulation may be insufficient, as capturing at least some kind of the brain’s inner causal dynamics is typically considered to be important for producing a first-person experience. The level at which that must be captured, however, is a subject of much debate. On the one hand, it is possible that capturing computational input–output relations of all the “relevant” components in the brain, or, in a similar view, the neural dynamics, would be sufficient. Others disagree with this computationalist view of the brain, believing that an emulation would need to have the same causal interactions between their components as in the biological brain, which might require a type of neuromorphic computing [131,132]. 

Whole-brain emulation does not necessarily require capturing every detail down to the level of atoms or quantum states. The concept of scale separation refers to the existence of distinct levels of organization within a system, where the behavior at one level can be described independently of the details at lower levels [132,133,134]. If there is sufficient scale separation between lower-level processes and the computationally relevant properties of the brain, it may be possible to emulate the brain at a higher level of abstraction, although this is currently an open question. The goal of whole-brain emulation, then, is to replicate not only the outputs of an individual’s brain (e.g., thoughts, memories, and behaviors), but also the causal relationships of the brain’s components that give rise to those outputs. If successful, a whole-brain emulation would match the biological brain’s outputs in every relevant way, after accounting for the range of variability expected if the biological brain experienced the same level of noise [130]. One proposed method for whole-brain emulation following brain preservation is to slice it into thin sections, scan each section at high resolution, and then use automated image analysis to reconstruct the 3D connectome (i.e., the wiring diagram of the brain’s neural connections) alongside biomolecule annotations. This biomolecule-annotated connectome data would be combined with a wealth of knowledge about how the brain works—far beyond what we know today—to create a detailed model of the entire brain, embedded in the rest of a particular body and environment at a particular point in time. If this model accurately captures all the relevant computational properties of the brain, some would argue that running it in a computer would be an emulation rather than merely a simulation, and would constitute revival of the person [135]. The emulated person could potentially be given a synthesized biological or robotic body to allow physical interaction with the world, or they could live in a purely digital world with a digital body. 

Whole-brain emulation as a revival strategy is one of the most polarizing topics in biostasis. Many argue that it should not count as revival at all, due to concerns about whether an emulated person would truly be the same individual, or whether it will ever be possible for a person’s individual conscious experience to be decoupled from the physical substrate of the brain that supports it [132]. However, others argue that if the brain of the emulated person is functionally identical to the biological brain, including having the same memories, personality, and medium of awareness, then they would share the same phenomenological experience, i.e., the same qualitative, first-person experience of consciousness [135,136]. Moreover, some expect that it is a more plausible way of achieving revival compared to the biological reconstruction of a preserved brain, especially in cases of extensive damage [137]. Survey data indicate that a substantial proportion of people interested in biostasis believe that destructive whole-brain emulation would represent a form of survival [6,138]. Although survey data do not mean that either side is correct, they do suggest that it is a relevant topic for the biostasis community to consider. Notably, even for those who think that whole-brain emulation would be philosophically acceptable, they may still not consider it desirable, for example due to concerns about potential maltreatment of the emulated person. A key point here is that there are many open questions in this area, requiring substantial further investigation. Some people choosing biostasis today may decide to defer to individuals in the future regarding the acceptability of whole-brain emulation, if it ever becomes technically feasible, because they will have much more knowledge about the brain, consciousness, the structure of future society, potential alternative revival methods, and other relevant topics [6]. 

For those who do not see emulation as an acceptable or desirable revival method, computational models built from high-resolution scans of preserved brains could still aid in guiding purely biological revival attempts. For example, a detailed nanometer-scale map of an individual’s brain could serve as a blueprint for repairing damaged tissue in an anatomically faithful way. Therefore, investment in research related to the simulation and emulation of whole brains and individual brain regions may be a fruitful approach for advancing the field of biostasis [139,140,141]. Initial progress and prototypes of whole-brain emulation could be performed in small organisms such as *C. elegans* or *Drosophila*. A key current bottleneck is gathering sufficient data on the biomolecule-annotated connectome of whole brains, which will require substantial further investment in basic science and technology. Performing research on whole-brain emulation of animals in an ethical manner is also a critical aspect of such research. We refer interested readers to previous reports on progress towards whole-brain emulation [130,142]. 

## 9. Conclusions

In this roadmap, we have attempted to outline the current state of research and future directions for the field of biostasis, with a focus on the scientific and technical aspects of brain preservation for potential future revival. This roadmap touches on several directions, including the development of both better chemical compounds and better delivery approaches for those chemicals. Ultimately, the development of improved biostasis methods will likely depend on a combination of both advanced preservation compounds and more effective delivery methods. Continued research into platforms and chemicals that could improve neural tissue preservation, alongside research into surgical techniques, cannulation methods, perfusion parameters, and long-term storage methods, would all be enormously valuable. One of the nice parts of contributing to a relatively small, historically stigmatized research field is that there is a wide ocean of potential research areas that would be fruitful to address. It is important to point out that the views described in this roadmap are just one perspective and that there may be differences of opinion among the people who developed the roadmap. Furthermore, the views described here are in many ways almost certainly incomplete or mistaken. The only problem is, of course, that we do not fully know which parts the incorrect ones are, much less what are the most impactful ones. Towards the goal of becoming less mistaken, we invite feedback from other members of the biostasis community and the scientific community at large on areas for improvement in this roadmap. 

Economic factors are crucial for any field of scientific research, and biostasis research is no exception. So far, funding for biostasis research has predominantly come from individuals and organizations with a vested interest in the field, such as cryonics companies and individual cryonicists. While this has allowed for some progress, the limited resources have constrained the scope and pace of research. To the best of our knowledge, there has never been specific funding awarded for biostasis research from any government agency. We believe that for the field to grow and reach its full potential, there is a need for funding from larger sources, such as government agencies, academic institutions, and philanthropic organizations without a specific focus on biostasis. However, this expansion of funding sources faces a significant challenge: many potential funders and members of the public are highly skeptical about the feasibility of biostasis. This creates a chicken-and-egg problem, where the field receives little funding because many people do not believe it is promising, but there has also been insufficient research to thoroughly investigate its potential.

Breaking this cycle will likely require innovative approaches to both research and public engagement. Possible strategies to address this include: (1) fostering collaborations between biostasis researchers and mainstream cryobiology or neuroscience labs to increase access to resources and the reliability of the research, (2) developing clearer roadmaps and milestones to demonstrate progress and potential, (3) engaging in more public outreach to address misconceptions and discuss ethical concerns, (4) exploring alternative funding models such as crowdfunding or decentralized science initiatives, and (5) leveraging funding for other research topics where synergies with problems relevant to biostasis exist and publishing resulting findings in reputable scientific journals. Additionally, emphasizing the potential spillover benefits of biostasis research to other fields, such as organ preservation for transplantation, treatment of acute brain injuries, or connectomics, could help attract broader interest and support. Overcoming these funding challenges will be critical for performing a thorough and objective examination of the true potential of biostasis technologies.

Most importantly, we call upon the scientific community to engage in collaborative, multidisciplinary research efforts to address the challenges outlined in this roadmap. By leveraging the expertise from individuals in diverse fields such as cryobiology, neuroscience, bioengineering, and computer science, we can accelerate progress in biostasis and work towards better understanding its potential. As we continue to advance our knowledge of the brain and develop sophisticated tools for preservation and restoration, we may uncover new insights that bring us closer to realizing the potential of this transformative technology. However, it is crucial to recognize that further research may also reveal significant limitations or challenges that could impact the feasibility of certain methods or the overall prospects of success for biostasis in the future. For example, we may learn that a given method of biostasis has a very low or nonexistent chance of revival, even in the distant future, or that all of the current methods are fundamentally limited in their ability to preserve the necessary information for successful revival. These potential research outcomes underscore the importance of maintaining a balanced and evidence-based perspective on the prospects of biostasis. Regardless of the ultimate feasibility of revival, it is our hope that this roadmap will serve as a catalyst for further research and innovation in the field, encouraging scientists, policymakers, funders, ethicists, and the public at large to engage in the critical discussions and research efforts necessary to thoroughly investigate biostasis as a potential option for those who are interested in it, while remaining grounded in the scientific realities that emerge from ongoing research.

## Figures and Tables

**Table 1:** Glossary of key terms used in this manuscript.

Term	Definition
Aldehyde fixation	A chemical preservation method using aldehyde-containing compounds like formaldehyde or glutaraldehyde to crosslink proteins and stabilize tissue structure.
Biostasis	The practice of preserving humans for the long-term with the intent of future recovery, if this ever becomes feasible.
Blood–brain barrier	A semipermeable border at the capillary endothelium that regulates the entrance and removal of many molecules between the bloodstream and the brain.
Connectome	A complete map of neural connections in a brain at the synaptic level.
CPA	Cryoprotective agent, a synonym for cryoprotectant.
Cryopreservation	The process of preserving biological tissue by cooling to very low temperatures.
Cryopreservation technology	A set of tools and methods used for low-temperature preservation, including cryoprotectants, other additives, and the methods of their administration.
Cryoprotectant	A chemical substance used to protect biological tissue from damage during the cryopreservation process by decreasing or preventing ice formation.
Glass transition temperature	The temperature range at which an a viscous liquid becomes a glass-like amorphous solid.
Molecular nanotechnology	In the context of biostasis, a hypothetical future technology involving atomic-scale engineering that could potentially repair preserved tissues in situ or reconstruct organisms from detailed scans.
Non-penetrating CPA	A cryoprotectant that acts extracellularly because it is too large to cross cell membranes.
Penetrating CPA	A cryoprotectant of low molecular mass (typically <100 g/mol) able to penetrate cell membranes and prevent intracellular ice formation.
Perfusion	The process of using pressure to drive the flow of a liquid through the vascular system of an organ or whole organism.
Vitrification	The process of converting a liquid material into a glass-like amorphous solid that is free from ice crystal formation. In biostasis, this involves a cryopreservation technology delivering high concentrations of cryoprotectants followed by cooling.
Whole-brain emulation	A hypothetical future technology involving the replication of an individual brain’s internal causal processes in a computer system, alongside a robotic or simulated body to enable interaction with the world.

**Table 2:** Brief description of upsides and downsides of contemporary methods used in biostasis.

Method	Upsides	Downsides
Pure cryopreservation	Aims to preserve tissue in a more native state without chemical fixation. Successful reversible cryopreservation has been achieved in brain tissue samples, small organs, and small organisms. Overlap with the organ banking field, which provides economic incentives for research.	Requires rapid cooling and warming rates which are challenging to achieve in large organs. Reversing cryopreservation damage is a major challenge for revival. Costs and complexity associated with long-term low-temperature storage.
Aldehyde fixation followed by cryopreservation	Excellent morphological preservation. Fixation can aid with ensuring adequate distribution of cryoprotectant prior to cooldown, thereby decreasing or preventing damage due to freezing. Combines two powerful preservation methods.	Chemical crosslinking alters biomolecular state of the tissue. Reversing fixation is a major challenge for revival. Costs and complexity associated with long-term low-temperature storage.
Aldehyde fixation with non-cryogenic storage	Excellent morphological preservation. Avoids challenges of cryopreservation procedure. Very low costs of long-term storage. Lower risk of damage if long-term care is temporarily disrupted, for example due to a pandemic or war.	Chemical crosslinking alters the biomolecular state of the tissue. Reversing fixation is a major challenge for revival. Long-term stability of tissue may be less than with cryogenic storage.

## Abbreviations

ASC—Aldehyde-stabilized cryopreservation; BBB—Blood–brain barrier; CPA—Cryoprotective agent; CT—Computed tomography; DMSO—Dimethyl sulfoxide; DSP—Dithiobis(succinimidyl propionate); iNOS—Inducible Nitric Oxide Synthase; ITS—Intermediate temperature storage; MRI—Magnetic resonance imaging; PARP—Poly adenosine diphosphate-ribose polymerase; S-Mix—Standardized Measure of Ischemic Exposure; SMT—S-methylisothiourea; Tg—Glass transition temperature.
